# Can Mitochondria DNA Provide a Novel Biomarker for Evaluating the Risk and Prognosis of Colorectal Cancer?

**DOI:** 10.1155/2017/5189803

**Published:** 2017-03-16

**Authors:** Han Shuwen, Yang Xi, Pan Yuefen

**Affiliations:** Huzhou Central Hospital, No. 198 Hongqi Road, Huzhou, Zhejiang 313000, China

## Abstract

Colorectal cancer (CRC) was one of the most frequent cancers worldwide. Accurate risk and prognosis evaluation could obtain better quality of life and longer survival time for the patients. Current research hotspot was focus on the gene biomarker to evaluate the risk and prognosis. Mitochondrion contains its own DNA and regulates self-replicating so that it can be as a candidate biomarker for evaluating the risk and prognosis of colorectal cancer. But there were already huge controversies on this issue. The review was to summarize current viewpoints of the controversial issues and described our understanding from the four aspects including mtDNA copy number, mitochondrial displacement loop, mtDNA variation, and mtDNA microsatellite instability, wishing the summary of the mtDNA in colorectal cancer could provide a meaningful reference or a valuable direction in the future studies.

## 1. Introduction

Colorectal cancer (CRC) was one of the common types of cancer with a high prevalence of cancer-related morbidity and mortality. Early diagnosis and accurate prognosis evaluation were conducive to reducing the negative health impact. Despite the continuous progress in diagnostic and therapeutic methods, it was still of great significance to seek for the novel biomarkers to evaluate the risk and prognosis of colorectal cancer. The promising biomarkers for this purpose were genes [1], noncoding RNAs [2, 3], proteins [4], gut microorganism [5], and so on. Seeking for more sensitive and accurate biomarker to evaluate the risk and prognosis of colorectal cancer was still striving on the way.

Organelles are the major components of the cells and play an important role in the occurrence and development of cancer cells. The variation of organelles deserves to be the biomarkers to evaluate the risk and prognosis of cancers. Mitochondria, as a vital organelle in almost all eukaryotic cells, have two major functions: oxidative phosphorylation and ATP production [6, 7]. Accumulating evidences suggested the mitochondria played a key role in the cell proliferation [8], cell apoptosis [9], and aging [10]. More, the mtDNA seemed to be involved in a wide range of diseases such as diabetes [11], obesity [12], AIDS [13], and cancers.

Mitochondrion contained its own DNA and regulated self-replicating. MtDNA (mitochondria DNA) was a circular, double-stranded DNA molecule. The outside loop was heavy strand (H-strand) and the inside was light strand (L-strand). The H-strand and the L-strand contained 28 and 9 genes, respectively. The mtDNA genes were lacking of intron coding regions and the displacement loop (D-loop) was the only one significant noncoding region of the mtDNA. The copies and point mutations of mtDNA varied with different individuals, organs ages, and diseases [14, 15]. Therefore, mtDNA copy number, mitochondrial displacement loop, and mtDNA variation play an important role in many mitochondrion-associated diseases.

The biological characteristics of mtDNA were as follows: (1) Matrilineal inheritance: matrilineal inheritance was a kind of genetic phenomenon that the genetic information passed down only through the female line. The mtDNA of fathers could not be transferred to the next generation, because the mtDNA of sperm could be specifically recognized and degraded by the ubiquitin hydrolases of ovum. (2) Heterogeneity: the mutations of mtDNA often occurred in the different mitochondria attributing to the mtDNA without the allelic gene like the nDNA (nuclear DNA). The results leaded to both of the mutant type and wild type in the same cells or tissues. (3) Threshold effect: a small number of mutant-type mtDNA in the cells could not affect the normal structure or function of the tissues because of the compensation action of wild-type mtDNA. The structure or function of the bodies or tissues could be affected only when the quantity of mutant-type mtDNA exceeded the threshold level [16, 17]. (4) Random distribution: the number and types of mtDNA of the somatic cells or ovum cells were randomly assigned to daughter cells when it passed to next generations [18].

An abundance of studies were concentrated on the association between the mtDNA and the CRC. But there were considerable debates on whether it could be utilized as valuable biomarkers of CRC. The goal of this review was to summarize current viewpoints of the controversial issues and to reveal new directions for future studies.

## 2. mtDNA Copy Number

The number of mitochondrial DNAs was different between individuals and between the tissues in human cells [19]. Changes of mitochondrial DNA (mtDNA) copy number were widely reported in CRCs. Both an increase and a decrease in mtDNA copy number had been reported. It remained controversial whether the mtDNA copy number could be utilized as valuable biomarkers to evaluate the risk and prognosis of CRCs. Here we gave a short summary on this subject.

The related literatures and research achievements of mtDNA copy number in CRCs were showed in Table 1. van Osch et al. found that mtDNA copy number was lower in CRC tissues and mtDNA copy number was lower in mutated BRAF and in microsatellite unstable (MSI) tumors, but it was higher in KRAS mutated tumors [20]. Wang et al. reported that the mtDNA copy number was lower in CRC tissues and it was correlated with lymph-node metastasis [21].

However, there were many contradictive views. Wang et al. supported that the mtDNA copy number increased in CRCs and it was a factor of poor prognosis [22]. The researches of Haja Mohideen et al. suggested that mtDNA copy number both increased and decreased in CRCs and no association of the mtDNA copy number change with OS or DFS [23]. More, latest studies in vitro showed both of p53 and TFAM expression could increase mtDNA copy number in CRC lines [24].

In the studies of free circulating mtDNA, Thyagarajan et al. investigated peripheral blood mtDNA copy number in 412 colorectal adenoma cases and 526 cancer-free controls and found there was no association between mitochondrial DNA copy number and colorectal adenomas by the analysis of unconditional logistic regression [25]. Some researchers put forward an idea about the U-shaped association between the relative mtDNA copy number in peripheral blood samples and colorectal cancer risk. Individuals with lower or higher relative mtDNA copy numbers were at increased risk of colorectal cancer [26]. In addition, Qu et al. found that when the leukocyte mtDNA content was higher, the prognosis in CRC patients was worse [27]. A prospective study found that the leukocytes mtDNA copy number among women who subsequently developed colorectal cancer was lower than that among women who remained cancer-free [28].

## 3. D-Loop

The D-loop (mitochondrial displacement loop) was an mtDNA noncoding region and it was as the major control region for the regulation of mitochondrial genome replication and expression as it contained the leading-strand origin of replication and the main promoter required for transcription. The entire length of D-loop was 1,124 bps according to the mitochondria database http://www.mitomap.org [29–31]. The D-loop was as the research hotspot and its complete or partial sequence had been widely investigated in CRCs. Whether the D-loop could be used to evaluate the risk and prognosis of colorectal cancer, the major point of the controversy was the D-loop mutations site and D-loop mutations frequency.

The related literatures and research results of D-loop in CRCs were showed in Table 2. Feng et al. investigated 44 colorectal cancer tissues and found the ratio of the methylation in D-loop region in colorectal cancer tissues was less than that in the noncancerous tissues [32]. Gao et al. found the similar results in 65 colorectal cancer patients [33]. Govatati et al. investigated the D-loop region of CRC patients in south Indian origin and put forward that D-loop sequence alterations were inherent risk factor for CRCs [34].

Furthermore, Bai et al. believed the minor haplotype of nucleotides 16290T in the D-loop region could be used as the biomarker to evaluate the prognosis for postoperative survival of CRCs [35]. And Legras et al. found the mutations of D310 sequence could be considered as a biomarker for early detection of CRCs [36].

Kassem et al. reported that the D-loop mutations frequency was higher in CRC and precancerous colorectal lesions [37], while Akouchekian et al. held that it was more likely to be epiphenomena [38]. But Chang et al. maintained that D-loop mutation occurred at significantly higher frequency in CRCs with p53 mutations [39]. Additionally, Chang et al. held it was not associated with prognosis of CRC patients [39], but Lièvre et al. believed that the D-loop mutation was a factor of poor prognosis in CRCs [40].

## 4. mtDNA Alterations

The technique of SNP (Single Nucleotide Polymorphism) was mostly used to investigate the mtDNA alterations in CRCs. The mitochondria DNA, as a biomarker for the diagnosis and prognosis of CRCs, varied from study to study. The controversy was focus on the site and frequency of mtDNA mutation in CRC.

The related literatures and research results of mtDNA alteration in CRCs were showed in Table 3. Numerous research studies showed the mtDNA mutation could be used as a biomarker for the diagnosis and prognosis of CRCs [65, 68, 69]; Chen et al. investigated 104 colorectal cancer patients in China and found the mtDNA proportion of the mtDNA 4977 bp deletion in CRC tissues was decreased [62]. And this view was supported by Dimberg et al. in Swedish and Vietnamese patients [55]. Furthermore, some mtDNA mutations such as mitochondrial subunit ND1 gene [61], G1576A (MT-RNR1) and G2975A (MT-RNR2) [70], and mitochondrial A12308G in tRNA (Leu(CUN)) [53] were considered as the valuable molecular targets. Besides, some research studies suggested mtDNA mutations heralded poor outcomes and tumorigenesis [44]. But several studies showed no association between mtDNA alterations and CRC risks [52, 64, 71].

## 5. mtDNA Microsatellite Instability

It had been already confirmed that MSI-H (high frequency MSI), MSI-L (low frequency MSI), and MSS (microsatellite stability) in human nuclear genome were significantly associated with the prognosis and recurrence of CRCs [72, 73]. The diagnostic value and prognostic evaluation value of mtMSI (mtDNA microsatellite instability) in CRCs remained undetermined, because the nMSI (nuclear microsatellite instability) had a unique value and significance in CRCs. We believed the mtMSI had a potential value on the risk and prognosis evaluation of colorectal cancer, although the researches in the field were relatively few.

The related literatures and research results of mtMSI in CRCs were showed in Table 4. Some studies suggested there was no association between the stromal mtMSI and stromal nMSI (nuclear microsatellite instability), and the stromal mtMSI was independent of stromal nuclear MSI (microsatellite instability) [66, 74]. A meta-analysis suggested mtMSI was higher in mCRC (metastatic colorectal cancer) patients with both of MSI and MSS in CRCs, but the prognosis was no correlation [51]. Some studies indicated mtMSI was higher in CRCs with Mn-SOD overexpression [67] and the (C)(n) repeat mtMSI was associated with tumor progression [66].

## 6. Discussion

The function of mitochondria was producing the adenosine triphosphate (ATP) via the oxidative phosphorylation system (OXPHOS) in normal physiology. It was generally acknowledged that the numbers of mitochondria showed an increase in high metabolism cells like heart muscle cells. Adequate amounts of energy were a necessary precondition for the uncontrolled rapid proliferation of cancer cells [50]. The number of mitochondria, such logic goes, was increased in the cancer tissues. However, the different researches got different or even contrary conclusions. The uncontrolled rapid proliferation, as the most important feature of cancer cells, was relative in vivo. Many factors including the pharmacological interventions, body's immune system, gene mutation, cancer cell heterogeneity, and nutritional deficiency restricted the proliferation of cancer cells. Thus, the mtDNA copy number as an independent biomarker to evaluate the risk and prognosis of colorectal cancer was inappropriate. But it might be more meaningful to act as the indicator of energy metabolism of cancers. Further studies should be focused on the association between the mtDNA copy number and energy metabolism, angiogenesis, and apoptotic cell proportion in vivo.

The expression of mitochondrial genes was in need of the assistance of the nuclear genes. The mitochondria retrograde cell signaling pathways illustrated that the mtDNA leaded to the changes of nDNA [75]. The number of aberrance mitochondria also affected the stress response and energy metabolism of the cancers. The variations of mutation sites and mutation frequency of mtDNA were found in the CRC tissues. There was a big disparity in the related literatures and research results because the study population had the different nationality, genders, ages, and living environment. We supported that the variations of mutation sites and mutation frequency of mtDNA could be used as auxiliary indicators to evaluate the risk and prognosis of CRC. But independent cohort studies with large sample size should be carried out to reduce the chance of confounding factors affecting investigation results.

The D-loop was the control region to regulate the replication and expression of the mitochondrial genome. More, the expression of the mitochondrial genome was controlled by nuclear DNA. The intertwined relationship between the mtDNA and nuclear DNA was unclear. There were large uncertainties about the association between the variations of mitochondrial genome and the risk and prognosis in CRCs. With the development of bioinformatics and gene sequencing technology, it might provide novel evidences for the mtDNA as the risk and prognosis factor in CRCs by decoding the molecular biological basis of tumorigenesis and progression and complex regulatory networks of interacting molecular components in the future. Besides, there were several interference factors of prognosis of CRCs including the treatment choices, patient's condition, the cancer stages, and biological behavior of cancers in the different researches and investigations. The large range, continuous, and dynamic surveillance of the changes of mtDNA was the further study directions to predict the value and role of the mtDNA.

Recent studies had shown that nuclear genome microsatellite instability was the significant predictor of prognosis CRCs [50, 72]. Much work remains to be done to make the definitive relationship between the mtMSI and nMSI clear. But that did not mean the mtMSI could not act as an interesting predictor to evaluate the prognosis of CRCs. The researches in this field were relatively insufficient. In addition, mtDNA damage and repair system was essential for maintaining genome integrity and stability. Its relevant factors such as Tfam, POLG, and OGG1 may provide clues for the risk and prognosis evaluation of CRCs in the further study.

Moreover, there existed more empirical evidences to support the hypothesis that mtDNA and mitochondrial dysfunction could act as initiator in carcinogenesis. Intensive researches demonstrated that one or several mechanisms such as the mtDNA variation, mitochondrial dynamics [76], excessive quantity increases, mitochondrial enzyme defects [77], and mitochondrial retrograde signaling [78] could bring about global genomes changes that altered cell morphology and function, such as ATP production, calcium homeostasis, integration of metabolism, and regulation of apoptosis, and eventually leaded to tumor formation [79]. The mtDNA and mitochondrial dysfunction plays a vital role in the initiation and progression of malignancies and targeting the mtDNA might be a potential strategy for the development of selective anticancer therapy.

The review was to summarize current viewpoints of the controversial issues and described our understanding from the four aspects including mtDNA copy number, mitochondrial displacement loop, mtDNA variation, and mtDNA microsatellite instability. In conclusion, we believed that the mtDNA could serve as a potential biomarker for evaluating the risk and prognosis of colorectal cancer after conducting more in-depth studies. The summary of the mtDNA provided a meaningful reference and a valuable direction for the future studies.

## Figures and Tables

**Table 1:** Association between the mtDNA copy number and the risk and prognosis in CRC.

Sample type	Findings	Potential utility	Association	Ref
Cancer, adenoma, and adjacent normal tissue from CRC patients (*n* = 56) and recurrent CRC (*n* = 16); colon mucosa samples from healthy subjects (*n* = 76).	MtDNA copy number in carcinoma tissues and adjacent tissues was lower than that in earlier resected adenoma tissues and MtDNA copy number in primary CRC tissues was lower than that in recurrent CRC tissues.	Prognosis evaluation	The association between mtDNA and survival seemed to follow an inverse U-shape with the highest HR observed in the second quintile of mtDNA copy number (HR = 1.70, 95% CI = 1.18, 2.44) compared to the first quintile.	[20]

Colorectal cancer tissues (*n* = 65) and the corresponding noncancerous tissues.	The mean relative mtDNA copy number in colorectal cancer tissues was higher than that in noncancerous tissues.	Risk evaluation	Increased in the CRC tissues.	[33]

Colorectal adenoma tissues (*n* = 412) and cancer-free controls (*n* = 526).	There was no association between logarithmically transformed relative mtDNA copy number and colorectal adenoma risk.	Risk evaluation	No association.	[25]

Colorectal cancer tissues (*n* = 274) and the corresponding noncancerous tissues.	The mtDNA copy number was increased in 60.4% of the CRC tissues. But there was no association between the mtDNA copy number and the prognosis.	Risk evaluation and prognosis evaluation	Increased in the CRC tissues but no association with the prognosis.	[23]

Colorectal cancer tissues (*n* = 9) and cancer-free controls (*n* = 9).	The mtDNA copy number was decreased in adenocarcinoma.	—	Decreased in adenocarcinoma.	[41]

Leukocyte CRC patients (*n* = 598).	Patients with high leukocyte mtDNA content showed worse overall survival (OS) and relapse-free survival (RFS).	Prognosis evaluation	Negative correlation between leukocyte mtDNA content and prognosis.	[27]

peripheral leukocytes from CRC (*n* = 444) and controls nested (*n* = 1,423).	Baseline mtDNA copy number was lower among women who subsequently developed colorectal cancer.	Risk evaluation	Lower in colorectal cancers.	[28]

Colorectal cancer tissues (*n* = 60) and the corresponding noncancerous tissues.	The mtDNA copy number was lower in CRC tissues and it was correlated with lymph-node metastasis. Patients with a lower mtDNA copy number tended to have lower 3-year survival.	Risk evaluation and prognosis evaluation	Decreased in the CRC tissues.	[42]

Colorectal cancer tissues (*n* = 44) and the corresponding noncancerous tissues.	The mtDNA copy number was increased in the CRC tissues and this increase was particularly noticeable in stages I and II.	Risk evaluation	Increased in the CRC tissues.	[43]

422 colorectal cancer cases (168 cases with prediagnostic blood and 254 cases with postdiagnostic blood) and 874 controls who were free of colorectal cancer among participants.	There was a U-shaped relationship between the relative mtDNA copy number and colorectal cancer risk. The lowest and highest quartiles of relative mtDNA copy numbers were 1.81 (1.13–2.89) and 3.40 (2.15–5.36), respectively.	Risk evaluation	U-shaped association between the relative mtDNA copy number and risk of colorectal cancer.	[26]

Colorectal cancer tissues (*n* = 54) and the corresponding noncancerous tissues.	The mtDNA copy number was increased in the CRC tissues.	Risk evaluation	Increased in the CRC tissues.	[44]

Colorectal cancer tissues (*n* = 25) and the corresponding noncancerous tissues.	The mtDNA copy number was decreased in CRCs.	Risk evaluation	Decreased in the CRC tissues.	[45]

The mitochondrial DNAs copy number was different between individuals and between the tissues in human cells. Changes of mtDNA copy number were widely reported in CRCs. The PCR was used as the most common method to detect the mtDNA copy number in the tissues. There were contradictory points of the association between the mtDNA copy number and the risk and prognosis in CRC.

**Table 2:** Association between the D-loop and the risk and prognosis in CRC.

Sample type	Findings	Potential utility	Targets	Ref
CRC tissues (*n* = 174) and cancer-free controls (*n* = 170)	The frequencies of 310*＇*C*＇* insertion (*p* = 0.0078), T16189C (*p* = 0.0097) variants, and 310*＇*C*＇*ins/16189C haplotype (*p* = 0.0029) in colorectal cancer were significantly higher than that in controls.	Risk evaluation	Nucleotide positions D310 and D16189	[34]

CRC tissues (*n* = 25)	The D310 mutation was found in 8/25 (32%) CRCs.	Risk evaluation	D310	[46]

Blood samples from 152 CRC patients	The minor haplotype of nucleotides 16290T and frequent haplotype of nucleotide 16298T in the hypervariable segment 1 (HV1) region of the D-loop were associated with high survival rate of CRCs. The nucleotide site of 16290 was identified as independent predictor for CRCs (RR, 0.379; 95% CI, 0.171–0.839; *p* = 0.017).	Risk evaluation and prognosis evaluation	16290T in HV1 region of the D-loop	[47]

CRC tissues (*n* = 65) and the corresponding noncancerous tissues	The methylation rate of the D-loop region in colorectal cancer tissues was decreased in clinicopathological stages III and IV comparing with that in stages I and II.	Prognosis evaluation	The methylation rate of D-loop	[33]

121 adenomas and seven adenocarcinomas and their corresponding germinal controls	The hypervariable sequence (HV-II) in the loop (D-loop) was significantly associated with the MT-CO2 gene, which represents the early molecular events in MAP (MUTYH-associated polyposis) tumorigenesis.	Risk evaluation and prognosis evaluation	HV-II	[48]

CRC tissues (*n* = 44) and the corresponding noncancerous tissues	The D-loop of most corresponding noncancerous tissues was methylated and the percentage was 79.5%, while this percentage was much smaller than that in the tumor tissues (11.4%).	Risk evaluation	The methylation rate of D-loop	[32]

Table 4 in the reference	The rate of D-loop mutations in CRCs was higher.	Risk evaluation	D-loop mutations frequency	[37]

Colorectal adenoma tissues (*n* = 40) and cancer-free controls (*n* = 150)	The rate of D-loop mutations in CRCs was higher.	Risk evaluation	D-loop mutations frequency	[38]

CRC tissues with p53 mutation (*n* = 88) and without p53 mutation (*n* = 106)	The rate of D-loop mutations was higher in CRCs with p53 mutation.	Risk evaluation	D-loop mutations frequency	[39]

64 colorectal adenomas (larger than 10 mm) and from 36 liver metastases of 15 metastatic CRC patients.	The mitochondrial D310 mutations frequency increased in the colorectal adenomas.	Risk evaluation	D310	[36]

Colorectal cancer tissues (*n* = 25) and the corresponding noncancerous tissues	40.0% (10/25) of the colorectal cancers harbored mutation(s) in the D-loop of mtDNA.	Risk evaluation	D-loop mutations frequency	[41]

Colorectal cancer tissues (*n* = 77) and the corresponding noncancerous tissues	9% (7/77) of the colorectal cancers harbored mutation(s) in the D-loop region of mtDNA.	Risk evaluation	D-loop mutations	[49]

CRC tissues (*n* = 35) and the corresponding noncancerous tissues	Polymorphisms located in hypervariable region I (67.9%) more than that in II (32.1%) of D-loop.	Risk evaluation and prognosis evaluation	Polymorphisms in the D-loop	[50]

CRC tissues (*n* = 95) and cancer-free controls (*n* = 95)	Thirty-two (34%) CRCs and 2 persons (2%) of the cancer-free controls harbored mutations in the D310 region of D-loop.	Risk evaluation	D310	[51]

The D-loop (mitochondrial displacement loop) was an mtDNA noncoding region and it was as the major control region for the regulation of mitochondrial genome replication and expression. The rate of D-loop mutations, the site of D-loop mutations, and the methylation rate of D-loop were investigated in CRCs. The table has summarized the current main points.

**Table 3:** Association between the mtDNA mutation and the risk and prognosis in CRC.

Sample type	Findings	Potential utility	Targets	Ref
CRC tissues (*n* = 50) and the corresponding noncancerous tissues. Control group comprised the blood samples from healthy persons (*n* = 100).	There was no association between the CAG repeat variants in the POLG gene and colorectal cancer risk.	Risk evaluation	CAG repeat variability in the POLG gene	[52]

CRC tissues (*n* = 30), control group comprised the blood samples from healthy persons (*n* = 100).	The A12308G, a polymorphic mutation in V-loop tRNA (Leu(CUN)), was found in 6 colorectal tumor tissues and 3 healthy controls.	Risk evaluation	A12308G alteration in tRNALeu (CUN)	[53]

60 Vietnamese and 138 Japanese CRCs tissues.	The frequency of mtDNA mutations in the Vietnamese CRCs was higher than that in the Japanese CRCs (19 of 44 [43%] versus 11 of 133 [9%], *p* < 0.001).	Risk evaluation	mtDNA mutations frequency	[54]

CRC tissues from 105 Swedish and 88 Vietnamese patients and the corresponding noncancerous tissues.	The mtDNA 4977 bp deletion was more frequent in normal tissues comparing with paired cancer tissues.	Risk evaluation	mtDNA 4977 bp deletion	[55]

CRC tissues (*n* = 21) and the corresponding noncancerous tissues.	The mtDNA mutation frequency in the CRC tissues was decreased comparing with adjacent nontumor tissues.	Risk evaluation	mtDNA mutations frequency	[56]

CRC tissues (*n* = 54) and the corresponding noncancerous tissues.	mtDNA haplogroup B4 was associated with colorectal cancer risk and poor outcomes.	Risk evaluation and prognosis evaluation	mtDNA haplogroup B4	[44]

Colon cancer (*n* = 86), rectal cancer (*n* = 43), and the corresponding noncancerous tissues.	Nonsynonymous mtDNA mutation was found in 57% of colon and rectal cancer	Risk evaluation	mtDNA mutations frequency	[57]

Three tissues (cancerous, paracancerous, and normal tissues), respectively, from 20 patients.	The frequency of mtDNA mutations: cancerous > paracancerous > normal tissues.	Risk evaluation	mtDNA mutations frequency	[58]

Hyperplastic polyps (*n* = 25), serrated adenomas (*n* = 32), traditional serrated adenomas (*n* = 19), and CRCs tissues (*n* = 138).	The mtDNA mutations frequency in carcinomas was not significantly higher than that in hyperplastic polyps and serrated adenomas.	Risk evaluation	mtDNA mutations frequency	[59]

CRC tissues (*n* = 30) and the corresponding noncancerous tissues.	T4216C mutation was in 8/30 CRC patients.	Risk evaluation	T4216C mutation	[60]

CRC tissues (*n* = 30) and the corresponding noncancerous tissues. Blood samples were from 25 healthy people.	The mtND1 gene mutations and polymorphisms were in 11 (45.8%) and 13 (54.2%) CRC tissues, respectively.	Risk evaluation	Mitochondrial subunit ND1 (mtND1)	[61]

CRC tissues (*n* = 104) and the corresponding noncancerous tissues.	The 4,977 bp deletion level decreased with the advancing of cancer.	Risk evaluation and prognosis evaluation	4,977 bp deletion in the major arch of the mitochondrial genome	[62]

Nuclear microsatellite instability in 38 rectal carcinomas and 25 sigmoid carcinomas.	ND1 microsatellite sequence alterations were detected in 2.6% rectal carcinomas. ND5 microsatellite sequence alterations were detected in 5.3% rectal carcinomas and 8% sigmoid carcinomas.	Risk evaluation	ND1 and ND5	[63]

2854 CRC cases and 2822 controls.	Five variants showed association with colon cancer. Three variants were associated with risk of CRC for MSI cases, with the strongest association for T4562C.	Risk evaluation	The T4562C sites	[64]

The mtDNA mutations frequency and mutations sites were investigated to explore the association between the mtDNA mutation and the risk and prognosis in CRC. But the association between mtDNA mutation and CRCs varied from study to study.

**Table 4:** Association between the mtDNA microsatellite instability and the risk and prognosis in CRC.

Sample type	Findings	Potential utility	Ref
CRC tissues (*n* = 100) and the corresponding noncancerous tissues.	The mtMSI was found in 30% of CRCs and it was associated with the poor prognosis.	Risk evaluation and prognosis evaluation	[65]

83 CRC tissues with a MSI tumor (including 39 patients with Lynch syndrome) and in 99mCRC patients with a microsatellite stable (MSS) tumor.	The mtMSI was high in mCRC patients with both MSI and MSS tumors, but no correlation with prognosis.	Risk evaluation and prognosis evaluation	[51]

The microdissected cancer epithelia and adjacent stromas of 48 sporadic CRCs.	The stromal mtMSI had no association with stromal nMSI or epithelial mtMSI.	Risk evaluation	[66]

CRC tissues (*n* = 35) and the corresponding noncancerous tissues.	mtMSI [310*＇*C*＇* insertion (*p* = 0.00001) and T16189C (*p* = 0.0007)] was increased in the CRC tissues.	Risk evaluation	[67]

Recent studies showed that nuclear genome microsatellite instability was the significant predictor of prognosis CRCs. But the association between the mtDNA microsatellite instability and the risk and prognosis needs to be further confirmed.
